# Mendelian randomisation study of the relationship between vitamin D and risk of glioma

**DOI:** 10.1038/s41598-018-20844-w

**Published:** 2018-02-05

**Authors:** Hannah Takahashi, Alex J. Cornish, Amit Sud, Philip J. Law, Ben Kinnersley, Quinn T. Ostrom, Karim Labreche, Jeanette E. Eckel-Passow, Georgina N. Armstrong, Elizabeth B. Claus, Dora Il’yasova, Joellen Schildkraut, Jill S. Barnholtz-Sloan, Sara H. Olson, Jonine L. Bernstein, Rose K. Lai, Minouk J. Schoemaker, Matthias Simon, Per Hoffmann, Markus M. Nöthen, Karl-Heinz Jöckel, Stephen Chanock, Preetha Rajaraman, Christoffer Johansen, Robert B. Jenkins, Beatrice S. Melin, Margaret R. Wrensch, Marc Sanson, Melissa L. Bondy, Clare Turnbull, Richard S. Houlston

**Affiliations:** 1 Division of Genetics and Epidemiology, The Institute of Cancer Research, London, UK; 2 Case Comprehensive Cancer Center, School of Medicine, Case Western Reserve University, Cleveland, Ohio USA; 3 Division of Biomedical Statistics and Informatics, Mayo Clinic College of Medicine, Rochester, Minnesota USA; 4 Department of Medicine, Section of Epidemiology and Population Sciences, Dan L. Duncan Comprehensive Cancer Center, Baylor College of Medicine, Houston, Texas USA; 5 School of Public Health, Yale University, New Haven, Connecticut USA; 6 Department of Neurosurgery, Brigham and Women’s Hospital, Boston, Massachusetts, USA; 7 Department of Epidemiology and Biostatistics, School of Public Health, Georgia State University, Atlanta, Georgia USA; 8 Duke Cancer Institute, Duke University Medical Center, Durham, North Carolina USA; 9 Cancer Control and Prevention Program, Department of Community and Family Medicine, Duke University Medical Center, Durham, North Carolina USA; 10 Department of Epidemiology and Biostatistics, Memorial Sloan Kettering Cancer Center, New York, New York USA; 11 Departments of Neurology and Preventive Medicine, Keck School of Medicine, University of Southern California, Los Angeles, California USA; 12 Department of Neurosurgery, University of Bonn Medical Center, Sigmund-Freud-Str. 25, 53105 Bonn, Germany; 13 Human Genomics Research Group, Department of Biomedicine, University of Basel, Basel, Switzerland; 14 Department of Genomics, Life & Brain Center, University of Bonn, Bonn, Germany; 15 Institute of Human Genetics, University of Bonn School of Medicine & University Hospital Bonn, Bonn, Germany; 16 Institute for Medical Informatics, Biometry and Epidemiology, University Hospital Essen, University of Duisburg-Essen, Essen, Germany; 17 Division of Cancer Epidemiology and Genetics, National Cancer Institute, Bethesda, USA; 18 Institute of Cancer Epidemiology, Danish Cancer Society, Copenhagen, Denmark, Rigshospitalet, University of Copenhagen, Copenhagen, Denmark; 19 Department of Laboratory Medicine and Pathology, Mayo Clinic Comprehensive Cancer Center, Mayo Clinic, Rochester, Minnesota USA; 20 Department of Radiation Sciences, Umeå University, Umeå, Sweden; 21 Department of Neurological Surgery, School of Medicine, University of California, San Francisco, California USA; 22 Institute of Human Genetics, University of California, San Francisco, California USA; 23 Sorbonne Universités UPMC Univ Paris 06, INSERM CNRS, U1127, UMR 7225, ICM, F-75013 Paris, France; 24 AP-HP, Groupe Hospitalier Pitié-Salpêtrière, Service de neurologie 2-Mazarin, Paris, France; 25 William Harvey Research Institute, Queen Mary University, London, UK; 26 Guys and St Thomas Foundation NHS Trust, Great Maze Pond, London UK; 27 Division of Molecular Pathology, The Institute of Cancer Research, London, UK

**Keywords:** Cancer epidemiology, CNS cancer, Risk factors

## Abstract

To examine for a causal relationship between vitamin D and glioma risk we performed an analysis of genetic variants associated with serum 25-hydroxyvitamin D (25(OH)D) levels using Mendelian randomisation (MR), an approach unaffected by biases from confounding. Two-sample MR was undertaken using genome-wide association study data. Single nucleotide polymorphisms (SNPs) associated with 25(OH)D levels were used as instrumental variables (IVs). We calculated MR estimates for the odds ratio (OR) for 25(OH)D levels with glioma using SNP-glioma estimates from 12,488 cases and 18,169 controls, using inverse-variance weighted (IVW) and maximum likelihood estimation (MLE) methods. A non-significant association between 25(OH)D levels and glioma risk was shown using both the IVW (OR = 1.21, 95% confidence interval [CI] = 0.90–1.62, *P* = 0.201) and MLE (OR = 1.20, 95% CI = 0.98–1.48, *P* = 0.083) methods. In an exploratory analysis of tumour subtype, an inverse relationship between 25(OH)D levels and glioblastoma (GBM) risk was identified using the MLE method (OR = 0.62, 95% CI = 0.43–0.89, *P* = 0.010), but not the IVW method (OR = 0.62, 95% CI = 0.37–1.04, *P* = 0.070). No statistically significant association was shown between 25(OH)D levels and non-GBM glioma. Our results do not provide evidence for a causal relationship between 25(OH)D levels and all forms of glioma risk. More evidence is required to explore the relationship between 25(OH)D levels and risk of GBM.

## Introduction

While glioma accounts for around 80% of malignant primary brain tumours^1^, thus far exposure to ionising radiation is the only well-established exogenous risk factor^2^. Vitamin D provides many health benefits, including increased bone strength and protection against autoimmune diseases and type 2 diabetes^3^. *In-vitro* studies have also suggested an anti-neoplastic role for vitamin D^4^. Several epidemiological studies have shown that vitamin D may indeed afford protection against the development of some cancers, including colon, prostate and breast cancer^5^. Associations in such observational studies do not however constitute evidence for a causal relationship and in some studies bias from confounding and reverse causation cannot be excluded.

Mendelian randomisation (MR) uses genetic markers as proxies for environmental exposures to determine the effect of the exposure on disease risk^6^. It therefore provides a strategy for establishing causal relationships where randomised control trials (RCTs) would involve either high cost or impractical study design. In the case of a possible relationship between vitamin D and glioma, the rarity of the cancer would limit any RCT to small sample sizes and would require lengthy follow up times.

We implemented two-sample MR analysis to examine the relationship between vitamin D and glioma risk in order to avoid the limitations of follow up time, reverse causation and confounding. Genotypes are randomly assigned at conception, thereby limiting confounding. Furthermore an individual’s genotype will always be established before the onset of disease, excluding the possibility of reverse causation. The genotype is in part equivalent to a lifetime vitamin D deficiency, and hence a lifetime follow-up time in a RCT. We determine the relationship between vitamin D and glioma risk using genetic variants associated with 25(OH)D levels, rather than measuring 25(OH)D levels directly.

Genetic variants identified by the Study of Underlying Genetic Determinants of Vitamin D and Highly Related Traits (SUNLIGHT) Consortium^7^ and the Canadian Multicentre Osteoporosis Study (CaMOS)^8^ were used as an instrumental variable (IV). We performed an MR analysis to test for a causal relationship between 25(OH)D levels and glioma, using summary data from a recent genome-wide association study (GWAS) meta-analysis performed by the Glioma International Case-Control Consortium (GICC)^9^.

## Methods

Two-sample MR was undertaken using GWAS data. Ethical approval was not sought for this specific project because all data came from the summary statistics of previously published GWAS, and no individual-level data were used.

### Genetic variant instruments for 25(OH)D level

**Figure 1:**
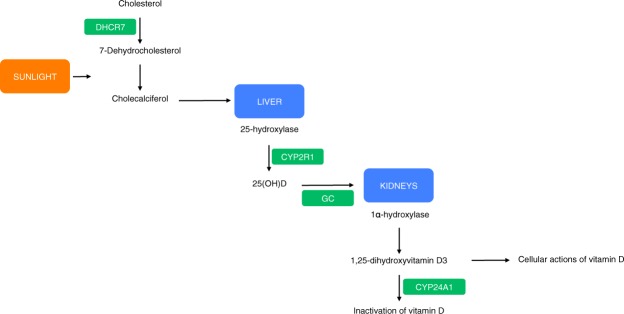
Effect of SNPs chosen as IVs on the vitamin D pathway. Genes that contain, or are in proximity to, variants chosen as IVs are highlighted green. *P* values for the association of these variants with 25(OH)D levels were 1.9 × 10^−109^ for *GC*, 2.1 × 10^−27^ for *DHCR7*, 3.3 × 10^−20^ for *CYP2R1*, and 6.0 × 10^−10^ for *CYP24A1*.

Genetic variants used as IVs were selected from the previously published SUNLIGHT study^7^. The SUNLIGHT Consortium GWAS identified four genetic variants associated with lowered 25(OH)D levels in 33,996 individuals of European descent from 15 cohorts. These variants were rs2282679 in *GC* (vitamin D binding carrier protein), rs10741657 near *CYP2R1* (converter of vitamin D to the active ligand for the vitamin D receptor), rs12785878 near *DHCR7* (7-dehydrocholesterol synthesis from cholesterol, a precursor to vitamin D) and rs6013897 in *CYP24A1* (degrader of active 1,25-dihydroxyvitamin D3 to inactive vitamin D)^10^. The roles of GC, CYP2R1, DHCR7 and CYP24A1 in the vitamin D pathway are shown in Fig. 1. Association estimates (*per*-allele log-ORs) for SNPs were taken from previously published studies, which used data from the CaMOS study, a population based cohort study of 2,347 Canadians, genotyped and assayed for 25(OH)D levels^8,10,11^. None of the SNPs were in linkage disequilibrium (*i.e*. r^2^ ≥ 0.001). For each SNP, we recovered the chromosome position, risk allele, genetic locus, F-statistic and association estimates (Table 1). Standard errors (SE) were calculated from F-statistics calculated by previous studies, which derive from the CaMOS cohort^11^. The risk allele was taken to be the 25(OH)D decreasing allele. Allele frequencies for these SNPs were compared between the 25(OH)D and glioma data sets to ensure that the effect estimates were recorded with respect to the same allele. This study calculated the variants to account for about 2% of the variation in circulating 25(OH)D levels, and have a combined F-statistic of 12.57^12^.

**Table 1:** Genetic variant instruments for 25(OH)D levels. EA, effect allele; NEA, non-effect allele; SE, standard error. Positions given using NCBI build 37. EA taken to be the 25(OH)D decreasing allele. Effect taken to be the per allele log OR effect on 25(OH)D.

SNP ID	Chr	Locus	Base pair position	EA glioma	NEA glioma	EA 25(OH)D	NEA 25(OH)D	Effect on 25(OH)D	SE	F-statistic
rs2282679	4	*GC*	72608383	G	T	G	T	−0.047	0.013	13.38
rs10741657	11	Near *CYP2R1*	14914878	G	A	G	A	−0.052	0.012	18.78
rs12785878	11	Near *DHCR7*	71167449	T	G	G	T	−0.056	0.013	18.29
rs6013897	20	*CYP24A1*	52742479	A	T	A	T	−0.027	0.015	3.13

### Glioma genotyping data

Association data between the four genetic variants and glioma were taken from the most-recent meta-analysis of GWAS in glioma^9^, which related >10 million genetic variants (after imputation) to glioma (Supplementary Table 1). This meta-analysis comprised eight GWAS datasets of individuals of European descent: FRE, GER, GICC, MDA, GliomaScan (NIH), UCSF-Mayo, UCSF and UK (Supplementary Table 2). All diagnoses were confirmed in accordance with WHO guidelines. Full quality control details are provided in previously published work^9^. Gliomas are heterogeneous and different tumour subtypes, defined in part by malignancy grade (for example, pilocytic astrocytoma World Health Organization (WHO) grade I, diffuse ‘low-grade’ glioma WHO grade II, anaplastic glioma WHO grade III and glioblastoma (GBM) WHO grade IV) can be distinguished^13^. To avoid diagnostic ambiguity and for simplicity we considered glioma subtypes as being either GBM or non-GBM.

### Statistical analyses

We examined the association between circulating 25(OH)D levels and glioma (including subtypes) using MR on summary statistics using the inverse variance weighted (IVW) and maximum likelihood estimation (MLE) methods, as described by Burgess *et al*.^14^. The combined ratio estimate ($\hat{\beta }$) of all SNPs associated with 25(OH)D levels on glioma risk was calculated under a fixed-effects model:

1 $$\hat{\beta }=\sum _{i=1}^{k}\frac{{X}_{k}{Y}_{k}{\sigma }_{Y}^{-2}}{{X}_{k}^{2}{\sigma }_{Y}^{-2}}$$

${X}_{k}$ is the association between SNP k with 25(OH)D levels, ${Y}_{k}$ is the association between SNP k and glioma risk with standard error ${\sigma }_{Y}$. The standard error of this association is given by:

2 $$se(\hat{\beta })=\sqrt{\sum _{i=1}^{k}\frac{1}{{X}_{k}^{2}{\sigma }_{Y}^{-2}}}$$

We also conducted a likelihood based analysis using the same genetic summary data^15^. For this maximum likelihood estimate, a bivariate normal distribution for the genetic associations was assumed, and the R function *optim* was used to estimate $\beta$. *SE* $(\beta )$ was calculated using observed information.

With the estimates from the two analyses calculated for each of the eight cohorts in the glioma data, we performed a meta-analysis under a fixed-effect model to derive final odds ratios (ORs) and confidence intervals (CIs)^16^.

To test whether the variants chosen as instruments were valid under MR assumptions, we examined the instruments for pleiotropy (multiple traits influenced by one gene) between the exposure and disease risk. This would be revealed as deviation from a linear relationship between SNPs and their effect size for 25(OH)D levels and glioma risk. We performed MR-Egger regression to test the average pleiotropic effect caused by the variants combined, as well as to provide a third association estimate between 25(OH)D level and glioma^17^. As per Dimitrakopoulou *et al*.^18^, we further evaluated the presence of horizontal pleiotropy by conducting stratified MR analyses using only the genetic variants influencing vitamin D synthesis (rs12785878, rs10741657) and vitamin D metabolism (rs2282679, rs6013897). rs12785878 has been associated with non-European status^10^ and we therefore also undertook a sensitivity analysis excluding rs12785878.

For each statistical test, we considered a global significance level of P < 0.05 as being satisfactory to derive conclusions. To assess the robustness of our conclusions, we imposed a conservative Bonferroni-corrected significance threshold of 0.017 (*i.e*. 0.05/3 tumour classifications).

The power of a MR investigation depends greatly on the proportion of variance in the risk factor that is explained by the IV. We therefore estimated study power to assess the strength of the results^19^. The detectable ORs at 80% power were 1.26 or 0.79 in the all glioma analysis, 1.34 or 0.75 in the GMB analysis and 1.35 or 0.74 in the non-GBM analysis. All power calculations were completed at a significance level of 0.05 and assumed the variants explained 2% of the total variance of 25(OH)D levels.

## Results

**Figure 2:**
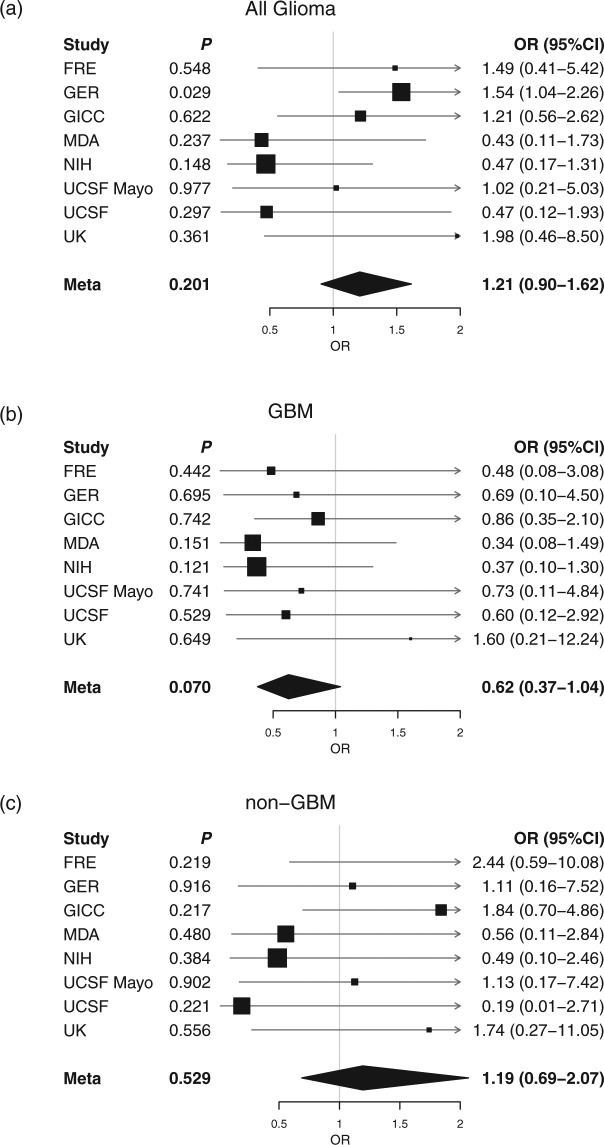
Individual cohort and meta-analysis ORs calculated using the IVW method. (**a**) All glioma, (**b**) GBM and (**c**) non-GBM glioma. Boxes are OR point estimates with area proportional to the weight of the study. Diamonds are overall summary estimates, with 95% CIs given by the width. Vertical line is null value (OR = 1.0).

**Figure 3:**
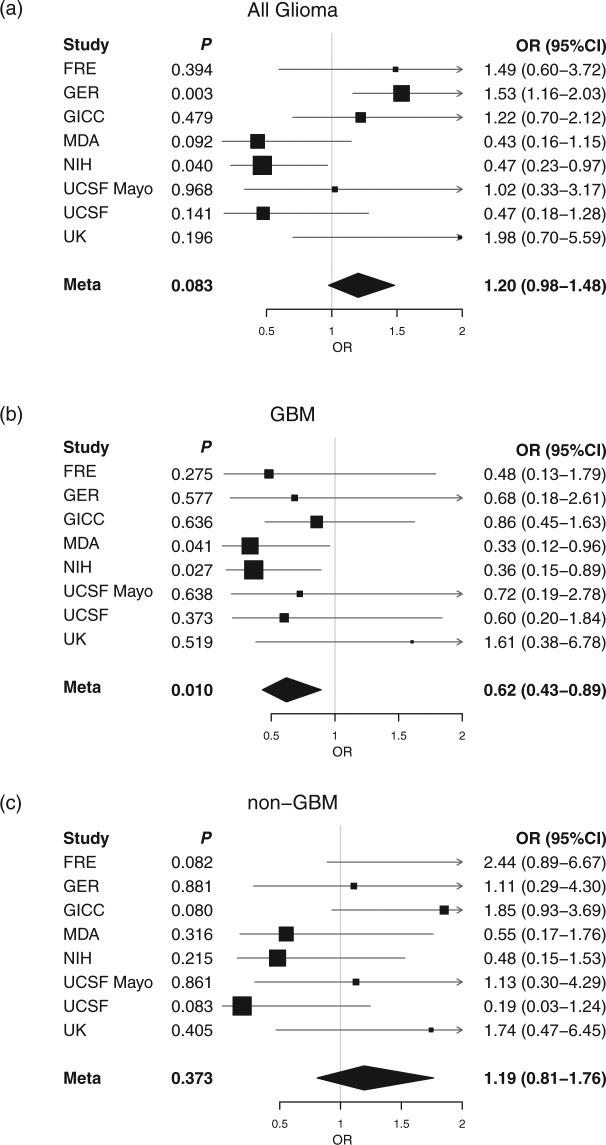
Individual cohort and meta-analysis ORs calculated using the MLE method. (**a**) All glioma, (**b**) GBM and (**c**) non-GBM glioma. Boxes are OR point estimates with area proportional to the weight of the study. Diamonds are overall summary estimates, with 95% CIs given by the width. Vertical line is null value (OR = 1.0).

The results of the IVW and MLE methods are summarised in Table 2. Results of the MR-Egger analysis are summarised in Table 3. Forest plots of all results from the IVW and MLE methods are shown in Figs 2 and 3. There was no evidence to support an association (*i.e. P* > 0.05) between circulating 25(OH)D levels and risk of all glioma using either the IVW (OR = 1.21, 95% CI = 0.90–1.62, *P* = 0.201) or MLE (OR = 1.20, 95% CI = 0.98–1.48, *P* = 0.083) methods. MR-Egger regression produced an intercept of −0.001 (95% CI = −0.019–0.017, *P* = 0.893) and therefore provided no evidence for pleiotropy amongst the genetic variants chosen as IVs (Supplementary Fig. 1). Hence there was no evidence of violation of MR assumptions.

**Table 2:** MR estimates between multi-SNP risk scores of 25(OH)D levels and all glioma, GBM and non-GBM glioma using the IVW and MLE methods. IVW, inverse-variance weighted; MLE, maximum likelihood estimation; SE, standard error; OR, odds ratio; CI, confidence interval; GBM, glioblastoma.

	IVW method				MLE method			
	β	SE(β)	OR (95% CI)	*P* value	β	SE(β)	OR (95% CI)	*P* value
All glioma	0.189	0.148	1.21 (0.90–1.62)	0.201	0.184	0.106	1.20 (0.98–1.48)	0.083
GBM	−0.471	0.261	0.62 (0.37–1.04)	0.070	−0.479	0.186	0.62 (0.43–0.89)	0.010
Non-GBM	0.177	0.281	1.19 (0.69–2.07)	0.529	0.177	0.199	1.19 (0.81–1.76)	0.373

**Table 3:** MR-Egger test results for 25(OH)D levels and all glioma, GBM and non-GBM glioma. CI, confidence interval; GBM, glioblastoma.

	MR Egger slope		MR Egger intercept	
	Estimate (95% CI)	*P* value	Estimate (95% CI)	*P* value
All Glioma	0.072 (−0.121–0.264)	0.466	−0.001 (−0.019–0.017)	0.893
GBM	−0.097 (−0.272–0.078)	0.279	−0.013 (−0.039–0.012)	0.307
Non-GBM	0.160 (−0.114–0.434)	0.253	−0.005 (−0.035–0.026)	0.768

We explored the possibility that a relationship between vitamin D and glioma may be subtype specific, considering GBM and non-GBM separately. We imposed a stronger significance threshold of *P* = 0.017 (*i.e*. 0.05/3), to correct for multiple testing. The MLE method identified an inverse relationship between 25(OH)D levels and risk of the GBM subtype, with an OR of 0.62 (95% CI = 0.43–0.89, *P* = 0.010). The IVW method provided a similar, but non-significant effect size (OR = 0.62, 95% CI = 0.37–1.04, *P* = 0.070). No evidence for an association between 25(OH)D levels and the non-GBM subtype was identified using either the IVW or MLE methods. MR-Egger regression provided intercepts of −0.013 (95% CI = −0.039–0.012, *P* = 0.307) for GBM and −0.005 (95% CI = −0.035–0.026, *P* = 0.768) for non-GBM, again providing no evidence of pleiotropy.

Stratified MR analyses using separate allelic scores for vitamin D synthesis and metabolism did not indicate the presence of horizontal pleiotropy (Supplementary Tables 3 and 4). To address the potential effects of population stratification, we undertook a MR sensitivity analysis excluding rs12785878, as this SNP has been associated with non-European status^10^ (Supplementary Table 5). Excluding rs12785878, the inverse relationship between 25(OH)D levels and risk of the GBM subtype identified by the MLE method remains significant (OR = 0.51, 95% CI = 0.33–0.80, *P* = 0.003), thereby providing no evidence that this association is a result of population stratification.

## Discussion

To our knowledge, this is the first MR study evaluating the effect of vitamin D on glioma risk undertaken. Overall our results do not provide evidence for an effect of vitamin D on risk of all forms of glioma. They do however raise the possibility for a protective role of vitamin D in GBM. While vitamin D and its metabolites have been shown to induce death of glioblastoma cells^20–22^, only one epidemiological study has investigated the relationship between pre-diagnostic levels of 25(OH)D and glioma risk^23^. Researchers found that higher levels of 25(OH)D were protective against high-grade glioma in men over the age of 56 (OR = 0.59), although the reverse trend was shown in men under the age of 56, albeit at a borderline-significant level^23^. Excluding the possibility of post hoc data mining, such paradoxical findings would support distinct aetiologies between the GBM and non-GBM subtypes, as has been suggested previously^9^.

Vital to the method of statistical analysis used herein is that none of the MR assumptions are violated. This requires that the variants chosen as IVs are (i) strongly associated with the exposure, (ii) are not associated with any confounding effects between exposure and outcome and (iii) are only associated with the outcome via the exposure. With regard to this study, the instruments chosen were associated with 25(OH)D levels at genome-wide significance levels. The MR-Egger test provided no evidence of horizontal pleiotropy, which we deemed sufficient to satisfy the third assumption. Furthermore, none of the four SNPs were in linkage disequilibrium (*i.e*. r^2^ ≥ 0.001) with any of the variants identified by Melin *et al*.^9^ as being in the risk region for glioma. With regard to confounding factors, few risk factors are known for glioma, so it was not possible to entirely rule out the possibility of unknown confounding factors causing statistical bias. However it should also be noted that all four SNPs lie either within or near genetic loci whose function in vitamin D physiology is well understood^7^, although a lack of knowledge of possible confounding factors means it was not possible to entirely rule out the possibility of confounding by unknown factors.

We acknowledge that a weakness of our study was in the small percentage of variability (around 2%) in 25(OH)D levels explained by the IV. Such a low value means any interpretation of these results as true indicators of the effect of total 25(OH)D levels on glioma risk are limited. This is quantified by the high ORs required for sufficient study power. Furthermore the study only accounts for circulating 25(OH)D levels and not for the action of 25(OH)D at the cellular level^11^. The genetic variants used as IVs in this MR analysis associate with 25(OH)D levels, rather than levels of the biologically active 1,25-dihydroxyvitamin D (1,25(OH)2D) and we therefore cannot explicitly comment on the relationship between 1,25(OH)2D and glioma. The low OR found in the GBM analysis should be noted however, given the fairly consistent indications of protective effects of 25(OH)D across all three methods. As is generally the case with MR, any findings should be viewed as a compliment to other future epidemiological studies, which test more robustly for associations between vitamin D and glioma and its subtypes.

In conclusion our MR analysis provides no evidence for an association between vitamin D and glioma, though findings raise the possibility of a potential association between vitamin D and GBM warranting further investigation.

## Electronic supplementary material

Supplementary Information

## Data Availability

Genotype data from the GICC GWAS are available from the database of Genotypes and Phenotypes (dbGaP; accession phs001319.v1.p1). Genotype data from the GliomaScan GWAS can also be accessed through dbGaP (accession phs000652.v1.p1). Data from the other studies are available upon request.
