# Publisher Correction: Mendelian randomisation study of the relationship between vitamin D and risk of glioma

**DOI:** 10.1038/s41598-019-43787-2

**Published:** 2019-05-23

**Authors:** Hannah Takahashi, Alex J. Cornish, Amit Sud, Philip J. Law, Ben Kinnersley, Quinn T. Ostrom, Karim Labreche, Jeanette E. Eckel-Passow, Georgina N. Armstrong, Elizabeth B. Claus, Dora Il’yasova, Joellen Schildkraut, Jill S. Barnholtz-Sloan, Sara H. Olson, Jonine L. Bernstein, Rose K. Lai, Minouk J. Schoemaker, Matthias Simon, Per Hoffmann, Markus M. Nöthen, Karl-Heinz Jöckel, Stephen Chanock, Preetha Rajaraman, Christoffer Johansen, Robert B. Jenkins, Beatrice S. Melin, Margaret R. Wrensch, Marc Sanson, Melissa L. Bondy, Clare Turnbull, Richard S. Houlston

**Affiliations:** 1 Division of Genetics and Epidemiology, The Institute of Cancer Research, London, UK; 2 Case Comprehensive Cancer Center, School of Medicine, Case Western Reserve University, Cleveland, Ohio USA; 3 Division of Biomedical Statistics and Informatics, Mayo Clinic College of Medicine, Rochester, Minnesota USA; 4 Department of Medicine, Section of Epidemiology and Population Sciences, Dan L. Duncan Comprehensive Cancer Center, Baylor College of Medicine, Houston, Texas USA; 5 School of Public Health, Yale University, New Haven, Connecticut USA; 6 Department of Neurosurgery, Brigham and Women’s Hospital, Boston, Massachusetts USA; 7 Department of Epidemiology and Biostatistics, School of Public Health, Georgia State University, Atlanta, Georgia USA; 8 Duke Cancer Institute, Duke University Medical Center, Durham, North Carolina USA; 9 Cancer Control and Prevention Program, Department of Community and Family Medicine, Duke University Medical Center, Durham, North Carolina USA; 10 Department of Epidemiology and Biostatistics, Memorial Sloan Kettering Cancer Center, New York, New York USA; 11 Departments of Neurology and Preventive Medicine, Keck School of Medicine, University of Southern California, Los Angeles, California USA; 12 Department of Neurosurgery, University of Bonn Medical Center, Sigmund-Freud-Str. 25, 53105 Bonn, Germany; 13 Human Genomics Research Group, Department of Biomedicine, University of Basel, Basel, Switzerland; 14 Department of Genomics, Life & Brain Center, University of Bonn, Bonn, Germany; 15 Institute of Human Genetics, University of Bonn School of Medicine & University Hospital Bonn, Bonn, Germany; 16 Institute for Medical Informatics, Biometry and Epidemiology, University Hospital Essen, University of Duisburg-Essen, Essen, Germany; 17 Division of Cancer Epidemiology and Genetics, National Cancer Institute, Bethesda, USA; 18 Institute of Cancer Epidemiology, Danish Cancer Society, Copenhagen, Denmark, Rigshospitalet, University of Copenhagen, Copenhagen, Denmark; 19 Department of Laboratory Medicine and Pathology, Mayo Clinic Comprehensive Cancer Center, Mayo Clinic, Rochester, Minnesota USA; 20 Department of Radiation Sciences, Umeå University, Umeå, Sweden; 21 Department of Neurological Surgery, School of Medicine, University of California, San Francisco, California USA; 22 Institute of Human Genetics, University of California, San Francisco, California USA; 23 Sorbonne Universités UPMC Univ Paris 06, INSERM CNRS, U1127, UMR 7225, ICM, F-75013 Paris, France; 24 AP-HP, Groupe Hospitalier Pitié-Salpêtrière, Service de neurologie 2-Mazarin, Paris, France; 25 William Harvey Research Institute, Queen Mary University, London, UK; 26 Guys and St Thomas Foundation NHS Trust, Great Maze Pond, London, UK; 27 Division of Molecular Pathology, The Institute of Cancer Research, London, UK

Correction to: *Scientific Reports* 10.1038/s41598-018-20844-w, published online 05 February 2018

The original version of this Article contained a typographical error in the spelling of the author Dora Il’yasova, which was incorrectly given as Dora ll’yasova. This has now been corrected in the PDF and HTML versions of the Article.

