# Supplementary material for: Mendelian randomisation study of the relationship between vitamin D and risk of glioma
Source: Sci Rep. 2018 Feb 5;8:2339. doi: 10.1038/s41598-018-20844-w (PMC5799201; doi:10.1038/s41598-018-20844-w)
Supplement: Supplementary file 1 — Supplementary Information [file 41598_2018_20844_MOESM1_ESM.pdf]

SUPPLEMENTARY INFORMATION

Mendelian randomisation study of the relationship between vitamin D and risk of glioma

Hannah Takahashi[1], Alex J. Cornish[1], Amit Sud[1], Philip J. Law[1], Ben Kinnersley[1], Quinn T. Ostrom[2], Karim Labreche[1], Jeanette E. Eckel-Passow[3], Georgina N. Armstrong[4], Elizabeth B. Claus[5,6], Dora Il'yasova[7,8,9], Joellen Schildkraut[8,9], Jill S. Barnholtz-Sloan[2], Sara H. Olson[10], Jonine L. Bernstein[10], Rose K. Lai[11], Minouk J. Schoemaker[1], Matthias Simon[12], Per Hoffmann[13,14], Markus M. Nöthen[14,15], Karl-Heinz Jöckel[16], Stephen Chanock[17], Preetha Rajaraman[17], Christoffer Johansen[18], Robert B. Jenkins[19], Beatrice S. Melin[20], Margaret R. Wrensch[21,22], Marc Sanson[23,24], Melissa L. Bondy[4], Clare Turnbull[1,25,26], Richard S. Houlston[1,27]

1. Division of Genetics and Epidemiology, The Institute of Cancer Research, London, UK
2. Case Comprehensive Cancer Center, School of Medicine, Case Western Reserve University, Cleveland, Ohio, USA
3. Division of Biomedical Statistics and Informatics, Mayo Clinic College of Medicine, Rochester, Minnesota, USA
4. Department of Medicine, Section of Epidemiology and Population Sciences, Dan L. Duncan Comprehensive Cancer Center, Baylor College of Medicine, Houston, Texas, USA
5. School of Public Health, Yale University, New Haven, Connecticut, USA
6. Department of Neurosurgery, Brigham and Women's Hospital, Boston, Massachusetts, USA
7. Department of Epidemiology and Biostatistics, School of Public Health, Georgia State University, Atlanta, Georgia, USA
8. Duke Cancer Institute, Duke University Medical Center, Durham, North Carolina, USA
9. Cancer Control and Prevention Program, Department of Community and Family Medicine, Duke University Medical Center, Durham, North Carolina, USA
10. Department of Epidemiology and Biostatistics, Memorial Sloan Kettering Cancer Center, New York, New York, USA
11. Departments of Neurology and Preventive Medicine, Keck School of Medicine, University of Southern California, Los Angeles, California, USA
12. Department of Neurosurgery, University of Bonn Medical Center, Sigmund-Freud-Str. 25, 53105 Bonn, Germany
13. Human Genomics Research Group, Department of Biomedicine, University of Basel, Basel, Switzerland
14. Department of Genomics, Life & Brain Center, University of Bonn, Bonn, Germany
15. Institute of Human Genetics, University of Bonn School of Medicine & University Hospital Bonn, Bonn, Germany
16. Institute for Medical Informatics, Biometry and Epidemiology, University Hospital Essen, University of Duisburg-Essen, Essen, Germany
17. Division of Cancer Epidemiology and Genetics, National Cancer Institute, Bethesda, USA
18. Institute of Cancer Epidemiology, Danish Cancer Society, Copenhagen, Denmark, Rigshospitalet, University of Copenhagen, Copenhagen, Denmark

19. Department of Laboratory Medicine and Pathology, Mayo Clinic Comprehensive Cancer Center, Mayo Clinic, Rochester, Minnesota, USA
20. Department of Radiation Sciences, Umeå University, Umeå, Sweden
21. Department of Neurological Surgery, School of Medicine, University of California, San Francisco, San Francisco, California, USA
22. Institute of Human Genetics, University of California, San Francisco, California, USA
23. Sorbonne Universités UPMC Univ Paris 06, INSERM CNRS, U1127, UMR 7225, ICM, F-75013 Paris, France
24. AP-HP, Groupe Hospitalier Pitié-Salpêtrière, Service de neurologie 2-Mazarin, Paris, France
25. William Harvey Research Institute, Queen Mary University, London, UK
26. Guys and St Thomas Foundation NHS Trust, Great Maze Pond, London, UK
27. Division of Molecular Pathology, The Institute of Cancer Research, London, UK

Supplementary Figure 1: Egger plots showing association estimates and intercepts for IVW and MR-Egger methods. (a) All glioma, (b) GBM and (c) non-GBM glioma. Individual SNP results (x axis, association between genetic variant and exposure; y axis, association between genetic variant and outcome) for MR-Egger represented as dots.

a)

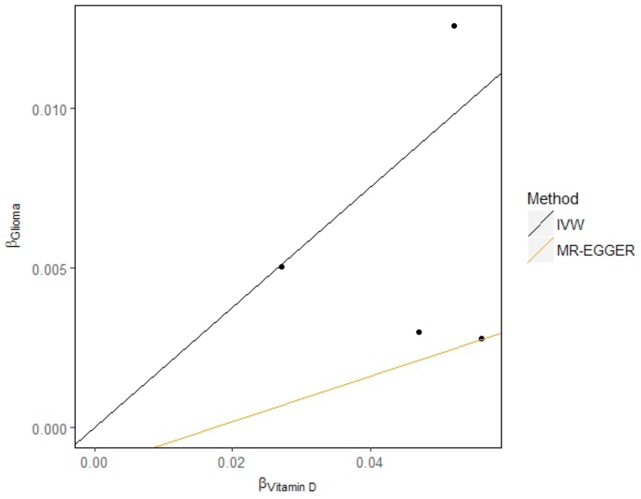

b)

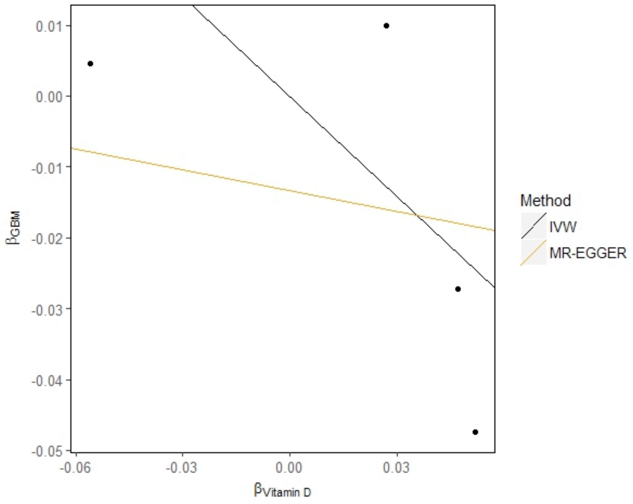

c)

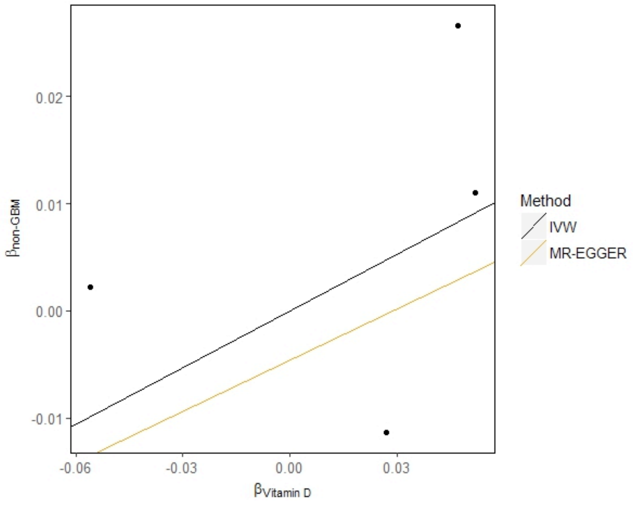

Supplementary Table 1: Summary data taken from the eight glioma GWAS cohorts for all glioma, GBM and non-GBM glioma.

		β (SE)							
	**SNP ID**	**FRE**	**GER**	**GICC**	**MDA**	**NIH**	**UCSF Mayo**	**UCSF**	**UK**
All glioma									
	rs2282679	-0.041014 (0.061236)	0.133969 (0.0722672)	0.0312392 (0.0370895)	-0.0201926 (0.0583145)	-0.0334083 (0.0502593)	-0.0852 (0.078)	0.026953 (0.0666535)	-0.083326 (0.068562)
	rs10741657	-0.043771 (0.084085)	-0.0231973 (0.0104501)	0.0471905 (0.0343338)	0.0227122 (0.065825)	0.0827191 (0.0459571)	0.0280 (0.0701)	0.0155846 (0.0626787)	-0.066564 (0.063921)
	rs12785878	0.010056 (0.091535)	0.0670007 (0.0731303)	0.103648 (0.0376908)	-0.184117 (0.0741074)	-0.0277272 (0.049879)	-0.0794 (0.0785)	-0.077447 (0.0701056)	-0.046863 (0.075283)
	rs6013897	0.069519 (0.096596)	-0.135191 (0.0804649)	-0.0238725 (0.0450309)	-0.0288026 (0.0643046)	0.0826986 (0.0555012)	-0.11586 (0.0891)	0.0171522 (0.0737805)	-0.0022402 (0.078694)
GBM									
	rs2282679	-0.04843 (0.087789)	0.194952 (0.091289)	0.0428872 (0.0426909)	-0.0248584 (0.0715876)	0.0531505 (0.0616334)	-0.0569 (0.0927)	0.052768 (0.0750633)	-0.050416 (0.086665)
	rs10741657	0.04091 (0.084085)	-0.0433447 (0.084627)	0.0838341 (0.0398032)	0.0227122 (0.065825)	0.136855 (0.0563093)	0.0299 (0.084)	-0.0159823 (0.070422)	-0.095132 (0.092758)
	rs12785878	-0.032299 (0.091535)	-0.0329982 (0.091797)	0.0898337 (0.0438104)	-0.184117 (0.0741074)	0.0587657 (0.0615237)	-0.1273 (0.0931)	-0.0522656 (0.0791768)	-0.061052 (0.10893)
	rs6013897	0.20486 (0.096596)	-0.220451 (0.0999851)	-0.0515566 (0.0521191)	-0.00551889 (0.0788602)	0.0656293 (0.0680447)	-0.1620 (0.1059)	0.0109484 (0.0830175)	0.078138 (0.11336)
Non-GBM									
	rs2282679	-0.037759 (0.066931)	0.0659646 (0.0925085)	0.0181318 (0.0467723)	-0.00983184 (0.0788454)	-0.13506 (0.0799106)	-0.1066 (0.0917)	-0.0557449 (0.125407)	-0.050416 (0.086665)
	rs10741657	-0.080648 (0.064092)	-0.00334915 (0.0856417)	-0.0131532 (0.0431055)	0.000852212 (0.0718896)	0.00882211 (0.0725762)	0.02888 (0.083)	0.137603 (0.119067)	-0.045619 (0.080722)
	rs12785878	0.029065 (0.070909)	0.0538005 (0.0946434)	0.111379 (0.0475173)	-0.136224 (0.0815494)	-0.146313 (0.0785833)	-0.0601 (0.0935)	-0.159781 (0.132543)	-0.036517 (0.095481)
	rs6013897	0.0094937 (0.075201)	-0.042059 (0.101502)	0.029179 (0.056441)	-0.0648037 (0.0868811)	0.184169 (0.0887083)	-0.0546 (0.1045)	0.0411433 (0.138721)	-0.063098 (0.10013)

Supplementary Table 2: Summary of the eight glioma GWAS cohorts.

Study series	Centre	Sampling	Cases	Controls
FRE	Groupe Hospitalier Pitié-Salpêtrière Paris	Patients with glioma were selected through the Service de Neurologie Mazarin, Groupe Hospitalier Pitié-Salpêtrière Paris. Individuals from the SU.VI.MAX (Supplementation en Vitamines et MinerauxAntioXydants) study were used as controls.	1,423	1,190
GER	University of Bonn	Comprised of patients who had undergone surgery between 1996 and 2008 for glioma at the Department of Neurosurgery, University of Bonn Medical Center. Control subjects were taken from three population studies: KORA (Co-operative Health Research in the Region of Augsburg); POPGEN (Population Genetic Cohort) and the Heinz Nixdorf Recall study.	846	1,310
GICC	GLIOGENE Consortium	Comprised of glioma cases and controls that were selected through Brigham and Women's Hospital (Boston, Massachusetts), Case Western Reserve University (Cleveland, Ohio), Columbia University (New York, New York), the Danish Cancer Society Research Centre (Copenhagen, Denmark), the Gertner Institute (Tel Hashomer, Israel), Duke University (Durham, North Carolina), the University of Texas MD Anderson Cancer Center (Houston, Texas), Memorial Sloan Kettering Cancer Center (New York, New York), the Mayo Clinic (Rochester, Minnesota), NorthShore HealthSystem (Chicago, Illinois), Umeå University (Umeå, Sweden), the University of California, San Francisco (San Francisco, California), the University of Southern California (Los Angeles, California), and the Institute of Cancer Research (London, United Kingdom). Cases had newly diagnosed glioma, and controls had no personal history of central nervous system tumors at the time of selection for the studies.	4,572	3,286
MDA	The University of Texas M.D. Anderson Cancer Center	Cases were ascertained between 1990 and 2008 through the MD Anderson Cancer Center, Texas. Controls were taken from the Cancer Genetic Markers of Susceptibility studies.	1,175	2,236
GliomaScan (NIH)	National Cancer Institute	Cases were newly diagnosed glioma (ICDO-3 codes 9380-9480 or equivalent), and controls were cancer-free.	1,653	2,725
UCSF-Mayo	Mayo Clinic	Comprised of Mayo cases, UCSF cases, and Mayo Clinic Biobank control data.	1,519	804
UCSF	University of California, San Francisco	Cases were adults with newly diagnosed, histologically confirmed glioma. Population-based cases who were diagnosed between 1991 and 2009 and who were residing in the six San Francisco Bay area counties were ascertained using the Cancer Prevention Institute of California's early-case ascertainment system. Clinic-based cases who were diagnosed between 2002 and 2012 were recruited from the UCSF Neuro-oncology Clinic, regardless of their place of residence. From 1991 to 2010, population-based controls from the same residential area as the population-based cases were identified using random digit-dialling and were frequency matched to population-based cases for age, gender and ethnicity. Between 2010 and 2012, all controls were selected from the UCSF general medicine phlebotomy clinic. Clinic-based controls were matched to clinic-based glioma cases for age, gender and ethnicity.	677	3,940
UK	INTERPHONE	Cases were ascertained through the INTERPHONE study. Controls were selected from the 1958 Birth Cohort.	631	2,699
Total			12,496	18,190

Supplementary Table 3: MR estimates between multi-SNP risk scores of 25(OH)D synthesis (rs12785878 and rs10741657) and all glioma, GBM and non-GBM glioma using the IVW and MLE methods.

	**IVW method**				**MLE method**			
	**β**	**SE(β)**	**OR (95% CI)**	***P* value**	**β**	**SE(β)**	**OR (95% CI)**	***P* value**
All glioma	0.207	0.161	1.23 (0.90-1.69)	0.197	0.200	0.117	1.22 (0.97-1.54)	0.086
GBM	-0.509	0.315	0.60 (0.32-1.11)	0.106	-0.514	0.225	0.60 (0.38-0.93)	0.023
Non-GBM	0.090	0.338	1.09 (0.56-2.12)	0.789	0.088	0.242	1.09 (0.68-1.75)	0.716

IVW, inverse-variance weighted; MLE, maximum likelihood estimation; SE, standard error; OR, odds ratio; CI, confidence interval; GBM, glioblastoma.

Supplementary Table 4: MR estimates between multi-SNP risk scores of 25(OH)D metabolism (rs2282679 and rs6013897) and all glioma, GBM and non-GBM glioma using the IVW and MLE methods.

	**IVW method**				**MLE method**			
	**β**	**SE(β)**	**OR (95% CI)**	***P* value**	**β**	**SE(β)**	**OR (95% CI)**	***P* value**
All glioma	0.088	0.382	1.09 (0.52-2.31)	0.817	0.090	0.271	1.09 (0.64-1.86)	0.741
GBM	-0.391	0.464	0.68 (0.27-1.68)	0.400	-0.392	0.330	0.68 (0.35-1.29)	0.234
Non-GBM	0.368	0.503	1.44 (0.54-3.87)	0.464	0.369	0.357	1.45 (0.72-2.91)	0.301

IVW, inverse-variance weighted; MLE, maximum likelihood estimation; SE, standard error; OR, odds ratio; CI, confidence interval; GBM, glioblastoma.

Supplementary Table 5: MR estimates between multi-SNP risk scores of 25(OH)D levels (excluding rs12785878, which has been associated with non-European status) and all glioma, GBM and non-GBM glioma using the IVW and MLE methods.

	**IVW method**				**MLE method**			
	**β**	**SE(β)**	**OR (95% CI)**	***P* value**	**β**	**SE(β)**	**OR (95% CI)**	***P* value**
All glioma	0.215	0.161	1.24 (0.90-1.70)	0.183	0.209	0.115	1.23 (0.98-1.55)	0.069
GBM	-0.666	0.319	0.51 (0.27-0.96)	0.037	-0.668	0.220	0.51 (0.33-0.80)	0.003
Non-GBM	0.285	0.344	1.33 (0.68-2.61)	0.407	0.285	0.244	1.33 (0.83-2.14)	0.242

IVW, inverse-variance weighted; MLE, maximum likelihood estimation; SE, standard error; OR, odds ratio; CI, confidence interval; GBM, glioblastoma.
